# Supplementary material for: Snacking on Whole Almonds for Six Weeks Increases Heart Rate Variability during Mental Stress in Healthy Adults: A Randomized Controlled Trial
Source: Nutrients. 2020 Jun 19;12(6):1828. doi: 10.3390/nu12061828 (PMC7353422; doi:10.3390/nu12061828)
Supplement: Supplementary file 1 [file nutrients-12-01828-s001.pdf]

Supplementary Materials

Supplemental Table S1. Nutritional composition of almonds and control snacks per 400 kcal isocaloric portions. Values given as 20% of the estimated average requirement for energy (2000 kcal) for adult women.

Nutrient	Almonds	Average of sweet and savory control snacks	Control snacks	
			Sweet	Savory
Energy, kcal	400	400	400	400
Protein, %E	13.4	9.8	9.8	9.8
Carbohydrate, %E	13.2	54.1	54.3	53.8
Starch, %E	-	38.7	24.5	52.9
Sugars, %E	2.3	15.1	29.7	0.4
Fibre, g	9.3	2.1	1.3	2.8
Fat, %E	73.6	36.5	35.8	37.1
SFA, g	5.6	12.8	12.6	12.8
MUFA, g	47.9	16.0	15.8	16.0
PUFA, g	16.0	5.6	5.4	5.9
Sodium, mg	<2.5	452.1	187.4	716.8
Potassium, mg	463.7	162.8	145.9	179.6
Calcium, mg	164.7	130.1	46.1	214.1
Magnesium, mg	181.7	19.0	15.4	22.6
Vitamin E, mg	14.3	1.3	1.1	1.4

Supplemental Figure S1. CONSORT Flow Diagram

Enrolment

Assessed for eligibility (n=294)

Excluded (n=187)
- Not meeting inclusion criteria (n=157)
- Declined to participate (n=10)
- Other reasons (n=20)

Assessed for eligibility after run-in (n=109)

Excluded (n=2)
- Not meeting inclusion criteria (non-compliance with the study protocols) (n=1)
- Declined to participate (time commitment issue) (n=1)

Randomized (n=107)

Allocation

Allocated to control group (n=51)
- Received allocated intervention (n=51)
- Did not receive allocated intervention (n=0)

Allocated to almond group (n=56)
- Received allocated intervention (n=56)
- Did not receive allocated intervention (n=0)

Follow-Up

Control group:
Lost to follow-up (n=0)
Discontinued intervention (n=0)

Almond group:
Lost to follow-up (n=0)
Discontinued intervention (gastrointestinal intolerance of almonds) (n=2)

Analysis

Control group:

Analyzed (n_{max}=51)

Included during analysis:
- Nutrient intakes: n=40
  (missing data are due to poor quality diet diaries or failure to complete by participants)
- *Blood pressure and heart rate variability*
  (missing data are due to poor quality read-outs and technological problems)
  - Clinical blood pressure: n=51
  - Resting NN, HR, SDNN, rMSSD and SD1/SD2: n=40
  - Resting HF, HFnu, and LF:HF: n=35
  - Stroop test NN and HR: n=35
  - Stroop test SDNN, rMSSD and SD1/SD2: n=36
  - Stroop test HF, HFnu and LF:HF: n=28
- *Body composition*
  - BMI: n=45, WC: n=49, Body fat: n=49
- *Circulating biomarkers*
  - HOMA-IR and glucose: n=48
  - Blood lipids: n=49

Almond group:

Analyzed (n_{max}=54)

Included during analysis:
- Nutrient intakes: n=40
  (missing data are due to poor quality diet diaries or failure to complete by participants)
- *Blood pressure and heart rate variability*
  (missing data are due to poor quality read-outs and technological issue)
  - Clinical blood pressure: n=54
  - Resting NN, HR, SDNN, rMSSD and SD1/SD2: n=43
  - Resting HF, HFnu, and LF:HF: n=40
  - Stroop test NN and HR: n=44
  - Stroop test SDNN, rMSSD and SD1/SD2: n=40
  - Stroop test HF, HFnu and LF:HF: n=35
- *Body composition*
  - BMI: n=50, WC: n=51, Body fat: n=52
- *Circulating biomarkers*
  - HOMA-IR and glucose: n=53
  - Blood lipids: n=53

Supplemental Table S2. Body composition, clinical blood pressure and circulating biomarkers of cardiometabolic risk following randomization to almond and control snacks.

	Control, n_{max} = 51[1]		Almonds, n_{max} = 54[1]		Main comparison between groups[3]
	Baseline[2]	Change	Baseline[2]	Change	
BMI, kg/m²	27.1 ± 4.4	-0.2 (-0.4, 0.0)	27.2 ± 4.5	0.1 (-0.1, 0.3)	0.2 (-0.1, 0.5)
WC, cm	93.3 ± 11.7	0.1 (-0.9, 1.2)	94.1 ± 12.2	-0.6 (-1.6, 0.5)	-0.7 (-2.2, 0.8)
Body fat, %	31.1 ± 7.7	-0.5 (-1.1, 0.0)	32.2 ± 7.7	0.3 (-0.3, 0.8)	0.8 (-0.0, 1.6)
cSBP, mmHg	127.8 ± 12.9	-5.2 (-7.9, -2.6)	127.3 ± 19.3	-6.3 (-8.9, -3.7)	-1.0 (-4.7, 2.6)
cDBP, mmHg	84.6 ± 7.9	-3.2 (-5.1, -1.3)	85.5 ± 10.6	-3.5 (-5.3, -1.6)	0.2 (-2.9, 2.4)
TC, mmol/L	5.26 ± 1.13	0.03 (-0.15, 0.20)	5.40 ± 0.93	-0.18 (-0.35, -0.02)	-0.21 (-0.45, 0.03)
TAG, mmol/L	1.17 (0.69)	-0.11 (-0.20, 0.01)	1.07 (0.73)	-0.08 (-0.17, 0.02)	0.03 (-0.11, 0.16)
Non-HDL, mmol/L	3.92 ± 1.16	0.11 (-0.04, 0.26)	4.00 ± 0.98	-0.11 (-0.25, 0.03	-0.22 (-0.42, -0.01)[4]
LDL, mmol/L	3.63 ± 1.16	0.15 (0.01, 0.30)	3.74 ± 0.91	-0.09 (-0.23, 0.05)	-0.25 (-0.45, -0.04)[4]
HDL, mmol/L	1.61 ± 0.45	0.04 (-0.04, 0.11)	1.66 ± 0.51	-0.04 (-0.11, -0.03)	-0.08 (-0.18, 0.03)
TC:HDL	3.45 ± 0.91	-0.04 (-0.15, 0.07)	3.47 ± 1.01	-0.03 (-0.14, 0.07)	0.00 (-0.15, 0.16)

Values of change and main comparison of the changes between groups are presented as mean (95% CI) generated from estimated marginal means from ANCOVA.

[1]Not all data were analysed due to technical problems and sample loss.

BMI: n = 45 (control) and 50 (almond).

WC: n = 49 (control) and 51 (almond).

Body fat: n = 49 (control) and 52 (almond).

TC, TAG, Non-HDL, LDL, HDL and TC:HDL: n = 49 (control) and 53 (almond).

[2]Median (IQR) for TAG data as they are non-normally distributed. Mean ± SD for other data that are normally distributed. Baseline biomarker values were not different between the two groups.

[3]ANCOVA, adjusted for baseline outcome value and baseline BMI (mean difference in change from baseline, almonds minus control); $P < 0.05$ indicating a significant difference.

[4]$p<0.05$ indicating a significant difference for values of mean difference between two groups.

BMI, body mass index; WC, waist circumference; cSBP, clinical systolic blood pressure; cDBP, clinical diastolic blood pressure; TC, total cholesterol; TAG, triglycerides; LDL, low-density lipoprotein cholesterol; HDL, high-density lipoprotein cholesterol.
